# High systemic immune-inflammation index predicts poor prognosis and response to intravesical BCG treatment in patients with urothelial carcinoma: a systematic review and meta-analysis

**DOI:** 10.3389/fonc.2023.1229349

**Published:** 2023-11-01

**Authors:** Wen Liu, Yixuan Zhang, Miaomiao Wang, Miao Wang, Qingya Yang

**Affiliations:** ^1^ Department of Urology, Beijing Hospital, National Center of Gerontology, Institute of Geriatric Medicine, Chinese Academy of Medical Sciences, Beijing, China; ^2^ Graduate School of Peking Union Medical College, Chinese Academy of Medical Sciences, Beijing, China; ^3^ School of Medicine, Qingdao University, Qingdao, Shandong, China; ^4^ Department of Urology, Qilu Hospital (Qingdao), Cheeloo College of Medicine, Shandong University, Qingdao, China

**Keywords:** urothelial carcinoma, meta-analysis, prognosis, systemic immune-inflammation index, Bacillus Calmette Guerin

## Abstract

**Background:**

The systemic immune-inflammation index (SII) has emerged as a promising marker predicting the prognosis of some cancers, while its role in urothelial carcinoma (UC) remains uncertain, especially in upper urinary tract urothelial carcinoma (UTUC). This meta-analysis aimed to investigate the association of SII with the prognosis of UC and the response to intravesical Bacillus Calmette-Guerin (BCG) therapy of non-muscle invasive bladder cancer (NMIBC).

**Methods:**

A systematic search in PubMed, Embase, Web of Science, and the Cochrane Library was performed to identify relevant studies. The extracted hazard ratios (HRs) and 95% confidence intervals (CIs) were used to evaluate the association between SII and overall survival (OS), cancer-specific survival (CSS), and recurrence-free survival (RFS) of patients with UC. Additionally, we pooled odds ratios (ORs) and 95% CIs to assess the relationship between SII and BCG response in patients with NMIBC. Subgroup and sensitivity analyses were performed to explore potential sources of heterogeneity.

**Results:**

Twenty studies comprising a total of 12,645 patients were eligible. This meta-analysis revealed that high SII levels independently increased the risk of OS (HR 1.55, 95%CI 1.25–1.92), CSS (HR 1.82, 95%CI 1.36–2.45), and RFS (HR 1.26, 95% CI 1.18–1.35) in patients with UC, including those with upper tract urothelial carcinoma. Additionally, elevated SII levels could predict a lower response to intravesical BCG treatment (OR 0.18, 95%CI 0.07–0.45) and higher disease recurrence (HR 1.61, 95%CI 1.31–1.98) in patients with NMIBC. Furthermore, elevated SII levels were positively associated with advanced age, lymphovascular invasion, hydronephrosis, and high tumor grade and stage (pT ≥ 3).

**Conclusions:**

Elevated preoperative SII levels are associated with poor survival outcomes in patients with UC, as well as worse response to BCG treatment in patients with NMIBC. Therefore, SII can serve not only as an independent prognostic predictor of patients with UC but also as a guide for BCG therapy in NMIBC.

**Systematic review registration:**

https://www.crd.york.ac.uk/prospero/display_record.php?ID=CRD42023409077, identifier CRD42023409077.

## 1 Introduction

Urothelial carcinoma (UC) is a prevalent malignancy, with 90-95% of cases occurring in the bladder and 5-10% occurring in the upper urinary tract (1). Although radical cystectomy (RC) is the standard treatment for patients with non-metastatic muscle-invasive bladder cancer (MIBC) and high-risk non-MIBC (NMIBC) patients, the 5-year survival rate after RC is only 66.9% (2, 3). Similarly, radical nephroureterectomy (RNU) with bladder cuff excision is the standard treatment of upper urinary tract urothelial carcinoma (UTUC), which also has a poor prognosis, with high rates of disease recurrence outside the bladder (28%) and mortality (23%) within 5 years after RNU (1, 4). Therefore, identifying the potential prognostic factors in UC is important for risk classification.

Inflammation response is a key player in promoting all stages of tumorigenesis, increasing the risk of cancer development (5, 6). Several immune inflammatory markers have been identified as potential indicators for tumor diagnosis and prognosis, such as neutrophil-lymphocyte ratio (NLR) and monocyte-lymphocyte ratio (MLR) (7, 8). Moreover, the systemic immune-inflammation index (SII), defined as neutrophils × platelets/lymphocytes, serves as a novel marker to reflect immune and inflammation conditions (9). Recent studies have suggested that high SII levels in UC were associated with poor prognosis (10–12), while others did not find a significant correlation (13–15).

According to the European Association of Urology (EAU) guidelines, transurethral resection of bladder tumor (TURBT) and intravesical Bacillus Calmette Guerin (BCG) treatment are recommended for intermediate- and high-risk NMIBC patients (16). BCG is the most efficacious intravesical therapy for reducing the recurrence of NMIBC and tumor progression (17, 18). However, in cases of BCG-unresponsive disease, patients are unlikely to respond to further BCG therapy and recommended to undergo RC (16). Therefore, predicting BCG failure may avoid ineffective BCG therapy and its adverse effects and facilitate timely RC or combined therapy. Several studies have reported that SII levels can predict the effectiveness of intravesical BCG therapy and disease recurrence in NMIBC patients receiving BCG (19–23). Based on these prior findings, the present meta-analysis aims to systematically estimate the association between SII and UC, including the prognostic value of SII in UC patients after surgery and its predictive value for response to intravesical BCG immunotherapy in NMIBC patients.

## 2 Materials and methods

### 2.1 Protocol

This meta-analysis was conducted according to the Preferred Reporting items for Systematic Review and Meta-analysis (PRISMA) guidelines and has been registered in PROSPERO (No. CRD42023409077).

### 2.2 Search strategy

To identify relevant available studies, we searched the PubMed, Embase, Web of Science, and the Cochrane Library databases from inception to March 5, 2023. The search terms included: (‘cancer’ or ‘carcinoma’ or ‘neoplasm’ or ‘tumor’) AND (‘urothelial’ or ‘ transitional’ or ‘bladder’ or ‘ upper tract’ or ‘ureter’ or ‘ renal pelvic’) AND (‘systemic immune-inflammation index’ or ‘SII’). Additionally, the reference sections of relevant articles were manually screened for eligible research.

### 2.3 Inclusion and exclusion criteria

Studies were included if they simultaneously met the following criteria: (1) articles reporting on the relationship between SII and the prognosis of UC after surgery or the response to intravesical BCG therapy, the formula of SII: SII = (neutrophils × platelets)/lymphocytes; (2) inclusion of a cut-off value for preoperative SII; (3) specific endpoints of interest, including overall survival (OS), cancer-specific survival (CSS), recurrence-free survival (RFS), or the response to intravesical BCG therapy; and (4) availability of hazard ratios (HRs) or odds ratios (ORs) with 95% confidence intervals (CIs) or the ability to calculate them from the data presented in the articles. Exclusion criteria were as follows: (1) reviews, case reports, conference abstracts, letters, and comments; (2) animal studies; (3) duplicate articles; (4) irrelevant studies; and (5) non-English articles. Two authors (WL and YXZ) independently selected the relevant literature, and disagreements were resolved by a third author (MMW).

### 2.4 Data extraction and quality assessment

We extracted data in three categories: (1) publication data: first author, publication year, age and country, (2) experimental data: cancer type, treatment, follow-up time, number of cases, and SII cut-off value, (3) outcome data: survival outcomes and BCG treatment response. Our meta-analysis considered different outcomes across included studies. For those examining the prognosis of patients who underwent RC, TURBT, or RNU, we focused primarily on OS, with CSS and RFS as secondary outcomes. Furthermore, for studies examining intravesical BCG treatment, we primarily evaluated the response to intravesical BCG therapy, with secondary outcomes including RFS and the area under the curve (AUC) for SII value to predict BCG failure. The quality of all included studies was evaluated using the Newcastle-Ottawa Scale (NOS), with studies scoring higher than six considered to be of high quality (**Supplementary Table 1**). Two authors (WL and YXZ) independently extracted data and assessed the quality of included studies, and discrepancies were resolved by a third author (MW).

### 2.5 Statistical analysis

HRs and 95% CIs were computed as the combined effect size to assess the relationship between pre-treatment SII and OS, CSS, and RFS. We also used pooled ORs and 95% CIs to determine the association of pre-treatment SII with response to BCG treatment, while the pooled AUCs and 95% CIs were used to predict BCG treatment failure. The heterogeneity among studies was qualitatively measured by Cochrane’s Q and I^2^ tests, with I^2^ > 50% and P < 0.10 indicating significant heterogeneity, and a random-effects model was used. Otherwise, a fixed-effects model was applied. Subgroup analysis was conducted to explore the potential sources of heterogeneity. Sensitivity analysis was performed to assess the effect of individual study data on survival outcomes. To evaluate the association between SII and clinicopathological factors, we extracted event and total numbers from two groups (high SII and low SII) to calculate pooled ORs and 95% CIs. Additionally, the publication bias was estimated by Begg tests. Statistical significance was defined as a 2-sided P-value < 0.05, and all analyses were conducted using R 4.1.1 (Lucent Technologies, Inc., Murray Hill, NJ, USA) and Stata version 15.0 (Stata Corp., College Station, TX).

## 3 Results

### 3.1 Study selection and characteristics

**Figure 1:**
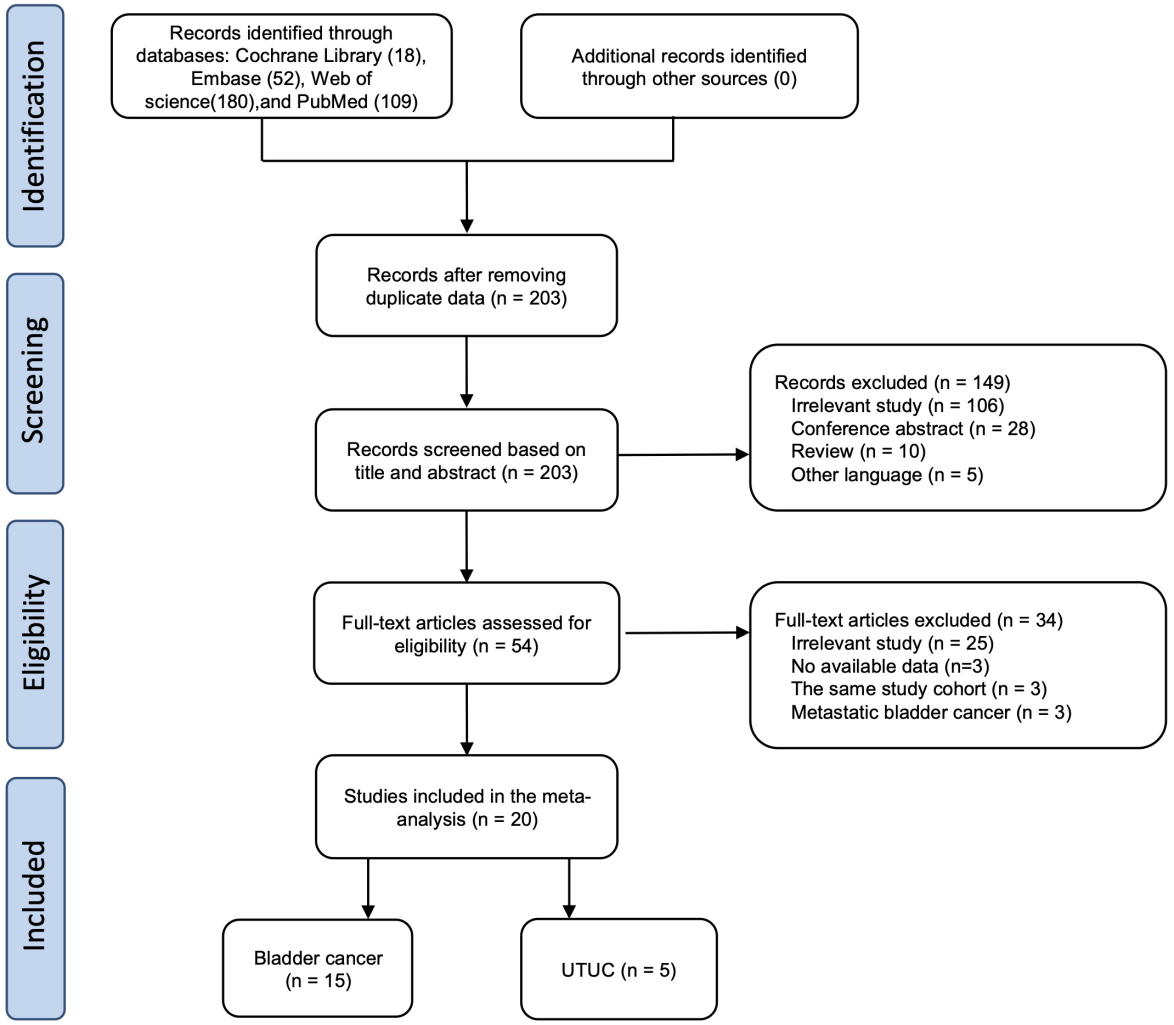
Flow chart of the study selection process.

The detailed screening process is shown in **Figure 1**. Initially, 359 articles were identified from three databases using specific search criteria. After removing duplicates, 203 articles remained for further screening. Upon evaluating the titles and abstracts, 54 articles were eligible for full-text screening. Out of the 54 studies, 34 were excluded for various reasons, including irrelevance to the study (25 studies), no available data (three studies), same study cohort (three studies), and focus on metastatic bladder cancer (three studies). Finally, 20 studies were considered eligible for quantitative synthesis (10–15, 19–32).

We included a total of 20 retrospective studies published between 2019 and 2022, which involved 12,645 patients. The SII cut-off values ranged from 276.69 to 1288. In terms of tumor types, nine studies focused on NMIBC, one on MIBC, five on mixed bladder cancer, and five on UTUC (**Table 1**). Specially, five studies explored the response to intravesical BCG therapy for patients with intermediate and high-risk NMIBC. Moreover, SII was evaluated in terms of its association with OS in 12 studies, CSS in nine studies, and RFS in 16 studies. All studies scored at least six on NOS, indicating their overall high quality. **Table 1** summarizes the main features of the included studies, such as tumor stage, lymph node involvement, lymphovascular invasion, and tumor grade.

**Table 1:** Summary of characteristics of the studies included in the meta-analysis.

Study	Author	Country	Multicenter	No. of cases	Follow-up (months)	Age* (years)	Tumor stage	Lymph node involvement	LVI	Grade (high)	Cancer type	Treatment	SII Cut-off	Cut-off selection	Survival outcome
2019	Zhang WT	China	No	209	NA	67.0	Tis, T1-T4	22	35	NA	MIBC+NMIBC	RC	507.7	X-tile	OS
2022	Zhang SY	China	No	725	OS:36; RFS:33.6	65.0	Tis, T1-T4	123	158	623	MIBC+NMIBC	RC	554.23	ROC	OS, RFS
2020	Ali Yılmaz	Turkey	No	152	16	66.0	T2-T4	72	84	97	MIBC	RC	768	ROC	OS, RFS
2022	Hasan Yilmaz	Turkey	No	241	20	65.0	T1-T4	89	105	NA	MIBC+NMIBC	RC	1228	X-tile	OS, RFS
2021	Yamashita	Japan	Yes	237	38	73.0	T0-T4	42	NA	NA	MIBC+NMIBC	RC	438	ROC	OS, CSS
2021	Grossmann	Austria	Yes	4335	42 (IQR 18-85)	67.0	Ta,Tis, T1-T4	1127	1475	4054	MIBC+NMIBC	RC	610	ROC	OS, RFS, CSS
2021	Zhao R	China	No	216	59.41 (IQR 2-89)	59.0	Ta,T1	0	NA	63	NMIBC	TURBT	276.69	ROC	RFS
2022	Wang C	China	Yes	149	42	63.73	Ta,T1	0	NA	79	NMIBC	TURBT	707.39	ROC	RFS
2021	Katayama	Austria	Yes	1117	64 (IQR 26-110)	67.0	Ta,Tis,T1	0	NA	488	NMIBC	TURBT	580	ROC	OS, RFS, CSS
2022	Li D	China	Yes	416	21 (IQR 14.75-32)	67.0	Ta,T1	0	NA	243	NMIBC	TURBT	505	X-tile	RFS
2020	Akan	Turkey	Yes	96	34.635 ± 14.7	62.8	Ta,T1	0	NA	87	NMIBC	TURBT+BCG	672.75	ROC	RFS
2020	Bi H	China	No	387	108 (IQR 5-191)	69.49	Ta,Tis,T1	0	31	263	NMIBC	TURBT+BCG	467.76	ROC	OS, CSS
2022	Li DX	China	No	197	15 (IQR 5-63)	64.17	Ta,T1	0	NA	142	NMIBC	TURBT+BCG	557	ROC	RFS
2021	Ke ZB	China	No	184	30 (IQR 13.0-47.0)	61.88	Ta,T1	0	NA	71	NMIBC	TURBT+BCG	439.83	ROC	RFS
2022	Liu P	China	No	183	46.8 ± 32.28	62.37	Ta,T1	0	NA	119	NMIBC	TURBT+BCG	514.47	ROC	RFS
2019	Jan H	China	No	424	33.8 (IQR 16.7-64.4)	69.3	Ta,Tis, T1-T4	25	115	402	UTUC	RNU	580	ROC	OS, RFS, CSS
2020	Zheng Y	China	Yes	525	52.0 (IQR 16.7)	67.6	T1-T4	41	79	392	UTUC	RNU	672.44	ROC	OS, RFS, CSS
2021	Chien	China	No	376	38	69.0	Ta,Tis, T1-T4	76	NA	300	UTUC	RNU	485	ROC	CSS, MFS, RFS
2021	Mori	Austria	Yes	2373	41 (IQR 20-60)	69.0	Ta,Tis, T1-T4	212	553	2002	UTUC	RNU	485	ROC	OS, RFS, CSS
2022	Kobayashi	Japan	No	103	NA	73.0	Ta,Tis, T1-T4	18	NA	76	UTUC	RNU	520	ROC	OS, CSS

*Median or Mean.

SII, systemic immune-inflammation index; MIBC, muscle invasive bladder cancer; NMIBC, non-muscle invasive bladder cancer; UTUC, upper urinary tract urothelial carcinoma; RC, radical cystectomy; TURBT, transurethral resection of bladder tumor; BCG, Bacillus Calmette-Guerin; RUN, radical nephroureterectomy; OS, overall survival; CSS, cancer-specific survival; RFS, recurrence-free survival; MF, metastasis-free survival; IQR, interquartile range; ROC, receiver operating characteristic; LVI, lymphovascular invasion; NA, not available.

### 3.2 Prognostic impact of SII on OS in patients with UC

**Figure 2:**
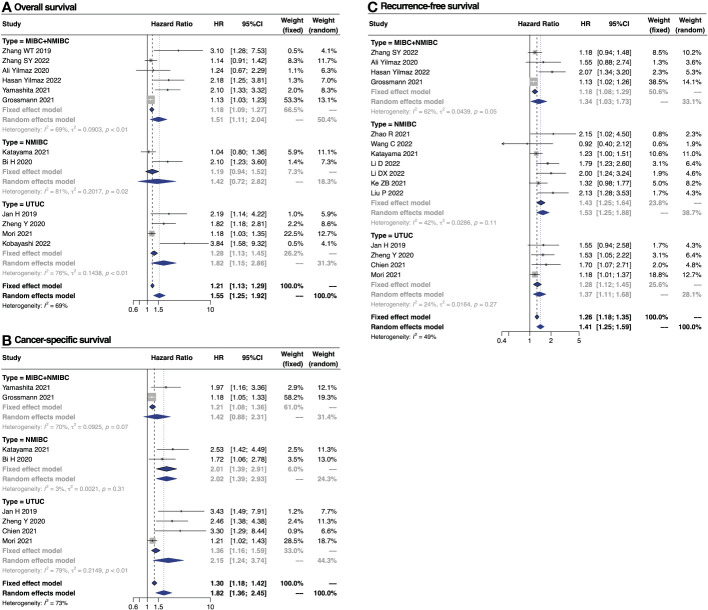
Forest plot showing the associations between SII and survival outcomes in patients with urothelial carcinoma. **(A)** OS; **(B)** CSS; **(C)** RFS.

Twelve studies, involving a total of 10,828 UC patients, examined the correlation between pre-treatment SII and OS (11, 12, 14, 15, 21, 24, 27–32). A random-effect model was employed to combine the results, revealing an adverse association between SII and OS (HR 1.55, 95% CI 1.25–1.92) (**Figure 2A**). Additionally, a subgroup meta-analysis based on tumor types showed that high SII increased the risk of all-cause mortality in patients with BC receiving RC (HR 1.51, 95% CI 1.11–2.04) and in those with UTUC receiving RNU (HR 1.82, 95% CI 1.15–2.86), but not in those with NMIBC receiving TURBT (HR 1.42, 95% CI 0.72–2.82), possibly due to the limited studies (only two) (**Figure 2A**). Given the high level of heterogeneity (I^2^ = 69%), we performed subgroup analyses and found that sample size might be a significant contributor to heterogeneity (both subgroups I^2^ = 0%). However, SII was still significantly related to OS in both subgroups of sample size, indicating the reliability of the results (**Table 2**).

**Table 2:** Subgroup analyses of OS, CSS, and RFS in patients with urothelial carcinoma.

Outcomes	Subgroups		No. of studies	Weight (%)	HR (95% CI)	*I^2^*	*P* for heterogeneity	Model
**OS**	Ethnicity	Asian	7	49.9	1.94 (1.43-2.63)	67%	<0.01	Random
		Caucasia	5	84.1	1.15 (1.07-1.23)	33%	0.2	Fixed
	Multicenter	No	7	46.3	1.88 (1.34-2.63)	66%	<0.01	Random
		Yes	5	53.7	1.30 (1.04-1.62)	66%	0.02	Random
	Sample size	<550	8	10	2.04 (1.67-2.50)	**0%**	0.57	Fixed
		>550	4	90	1.14 (1.06-1.22)	**0%**	0.86	Fixed
	Cancer type	MIBC	6	50.4	1.51 (1.11-2.04)	69%	<0.01	Random
		NMIBC	2	18.3	1.42 (0.72-2.82)	81%	0.02	Random
		UTUC	4	31.3	1.82 (1.15-2.86)	76%	<0.01	Random
	Cut-off value	<550	5	36.3	2.01 (1.32-3.08)	79%	<0.01	Random
		>550	7	63.7	1.33 (1.08-1.64)	56%	0.03	Random
	NOS	<9	6	48	1.73 (1.20-2.49)	72%	<0.01	Random
		=9	6	52	1.45 (1.09-1.94)	68%	<0.01	Random
**CSS**	Ethnicity	Asian	5	10.9	2.20 (1.67-2.89)	**0%**	0.54	Fixed
		Caucasia	3	49.4	1.40 (0,96-2.03)	69%	0.04	Random
	Multicenter	No	5	72.7	1.62 (1.16-2.27)	73%	<0.01	Random
		Yes	3	5.6	2.21 (1.51-3.23)	29%	0.24	Fixed
	Sample size	<550	5	10.9	2.20 (1.67-2.89)	**0%**	0.54	Fixed
		>550	3	49.4	1.40 (0.96-2.03)	69%	0.04	Random
	Cancer type	MIBC	2	31.4	1.42(0.88-2.31)	70%	0.07	Random
		NMIBC	2	6	2.01 (1.39-2.91)	3%	0.31	Fixed
		UTUC	4	44.3	2.15 (1.24-3.74)	79%	<0.01	Random
	Cut-off value	<550	4	50.4	1.65 (1.15-2.37)	62%	0.05	Random
		>550	4	49.6	2.04 (1.24-3.38)	83%	<0.01	Random
	NOS	<9	3	36.6	1.91 (1.03-3.55)	78%	0.01	Random
		=9	5	63.4	1.83 (1.27-2.64)	76%	<0.01	Random
**RFS**	Ethnicity	Asian	10	28	1.46 (1.28-1.65)	17%	0.29	Fixed
		Caucasia	5	72	1.19 (1.10-1.28)	50%	0.09	Fixed
	Multicenter	No	9	24.7	1.48 (1.29-1.69)	27%	0.21	Fixed
		Yes	6	75.3	1.19 (1.10-1.29)	35%	0.17	Fixed
	Sample size	<550	11	23.1	1.64 (1.42-1.88)	**0%**	0.69	Fixed
		>550	4	76.9	1.16 (1.08-1.25)	**0%**	0.89	Fixed
	Cancer type	MIBC	4	33.1	1.34 (1.03-1.73)	62%	0.05	Random
		NMIBC	7	23.8	1.43 (1.25-1.64)	42%	0.11	Fixed
		UTUC	4	25.6	1.28 (1.12-1.45)	24%	0.27	Fixed
	Cut-off value	<550	6	39.1	1.52 (1.23-1.89)	53%	0.06	Random
		>550	9	68.3	1.22 (1.12-1.32)	41%	0.09	Fixed
	NOS	<9	8	49.7	1.55 (1.26-1.90)	55%	0.03	Random
		=9	7	61.3	1.21 (1.11-1.31)	27%	0.22	Fixed

OS, overall survival; CSS, cancer-specific survival; RFS, recurrence-free survival; CI, confidence interval; HR, hazard ratio; MIBC, muscle invasive bladder cancer; NMIBC, non-muscle invasive bladder cancer; UTUC, upper urinary tract urothelial carcinoma; NOS, Newcastle-Ottawa Quality Assessment Scale.

Bold value: There was no heterogeneity in this subgroup meta-analysis.

### 3.3 Prognostic impact of SII on CSS in patients with UC

Nine studies, involving a total of 9501 UC patients, were investigated to determine the association between SII and CSS (10–12, 21, 24, 27–30). The pooled outcome suggested that high SII was significantly associated with poor OS (HR 1.82, 95% CI 1.36–2.45) (**Figure 2B**). Furthermore, subgroup analysis based on tumor types revealed that high SII increased the risk of cancer-specific mortality in patients with UTUC undergoing RNU (HR 2.15, 95% CI 1.24–3.74) and in those with NMIBC undergoing TURBT (HR 2.01, 95% CI 1.39–2.91), but not in those with BC undergoing RC (HR 1.42, 95% CI 0.88–2.31) (**Figure 2B**). Notably, only two studies were included in the meta-analysis of NMIBC and mixed BC, respectively. Due to the significant heterogeneity (I^2^ = 73%), we performed subgroup analyses and found that sample size and ethnicity might be the source of heterogeneity (**Table 2**).

### 3.4 Prognostic impact of SII on RFS in patients with UC

Sixteen studies, including a total of 10,757 UC patients, reported on the prognostic value of SII in RFS (10–15, 19, 20, 22–26, 28, 29, 31). The pooled analysis revealed that patients with high SII tended to have poor RFS (HR 1.26, 95% CI 1.18–1.35), based on a fixed effect model (I^2^ = 49%, P = 0.02) (**Figure 2C**). Moreover, subgroup analyses showed that high SII increased the risk of disease recurrence in patients with BC receiving RC (HR 1.34, 95% CI 1.03–1.73), in those with NMIBC receiving TURBT (HR 1.43, 95% CI 1.25–1.64), and in those with UTUC receiving RNU (HR 1.28, 95% CI 1.12–1.45), with relatively low heterogeneity (MIBC, I^2^ = 62%; NMIBC, I^2^ = 42%; UTUC, I^2^ = 24%) (**Figure 2C**). Additionally, the two subgroups with different sample sizes showed a significant relationship between high SII and poor RFS, with low heterogeneity respectively (both subgroups I^2^ = 0%) (**Table 2**).

### 3.5 Association between SII and BCG treatment response in patients with NMIBC

**Figure 3:**
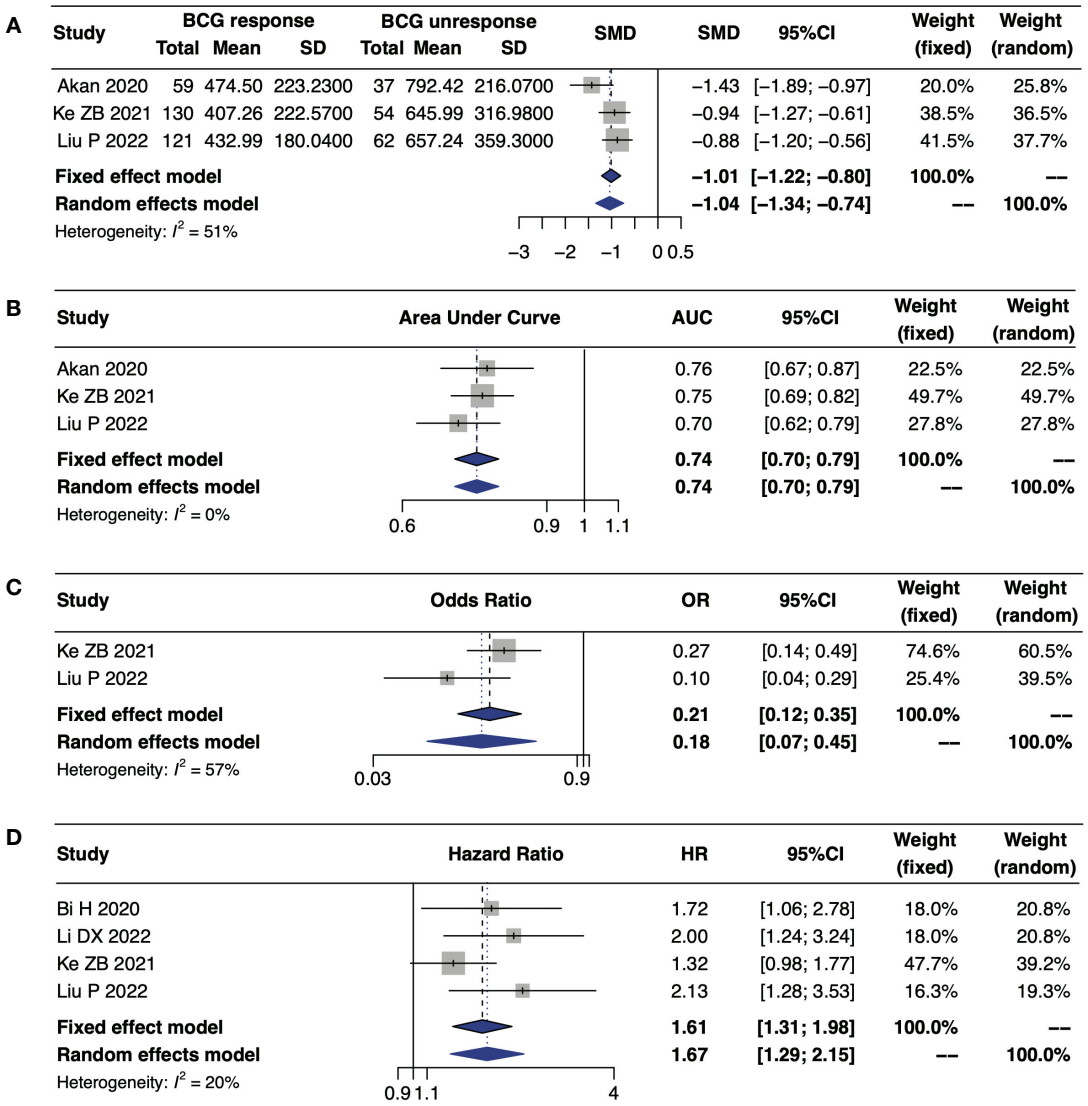
Forest plot of the predictive value of SII for patients with NMIBC undergoing TURBT and intravesical BCG therapy. **(A)** the SII levels in BCG-responsive and BCG-unresponsive groups; **(B)** AUC for predicting BCG response; **(C)** the value of SII predicting response to intravesical BCG therapy based on multivariable logistic regression analysis; **(D)** RFS for individual studies.

Five studies investigating the predictive role of SII on intravesical BCG response in patients with NMIBC (19–23). Compared to the BCG-unresponsive group, SII level was significantly lower in the BCG-responsive group (standardized mean difference [SMD] -1.04, 95% CI -1.34–0.74) (**Figure 3A**). Additionally, three studies reported the AUC of SII for BCG response, and the combined AUC was 0.74 (95% CI 0.70–0.79, I^2^ = 0%), indicating that SII was a reliable predictor of BCG treatment response (19, 22, 23) (**Figure 3B**). Ke ZB et al. and Liu P et al. revealed that low SII levels were independently linked to a positive BCG treatment response, as determined through multivariate logistic regression analysis (pooled OR 0.18, 95% CI 0.07–0.45, I2 = 57%) (**Figure 3C**). Notably, this meta-analysis incorporated data from only two studies. Furthermore, four studies suggested that high SII levels significantly increased the risk of disease recurrence in patients with NMIBC (pooled HR 1.61, 95% CI 1.31–1.98, I^2^ = 20%) (**Figure 3D**).

### 3.6 Associations between SII and clinicopathological features of patients with UC

**Figure 4:**
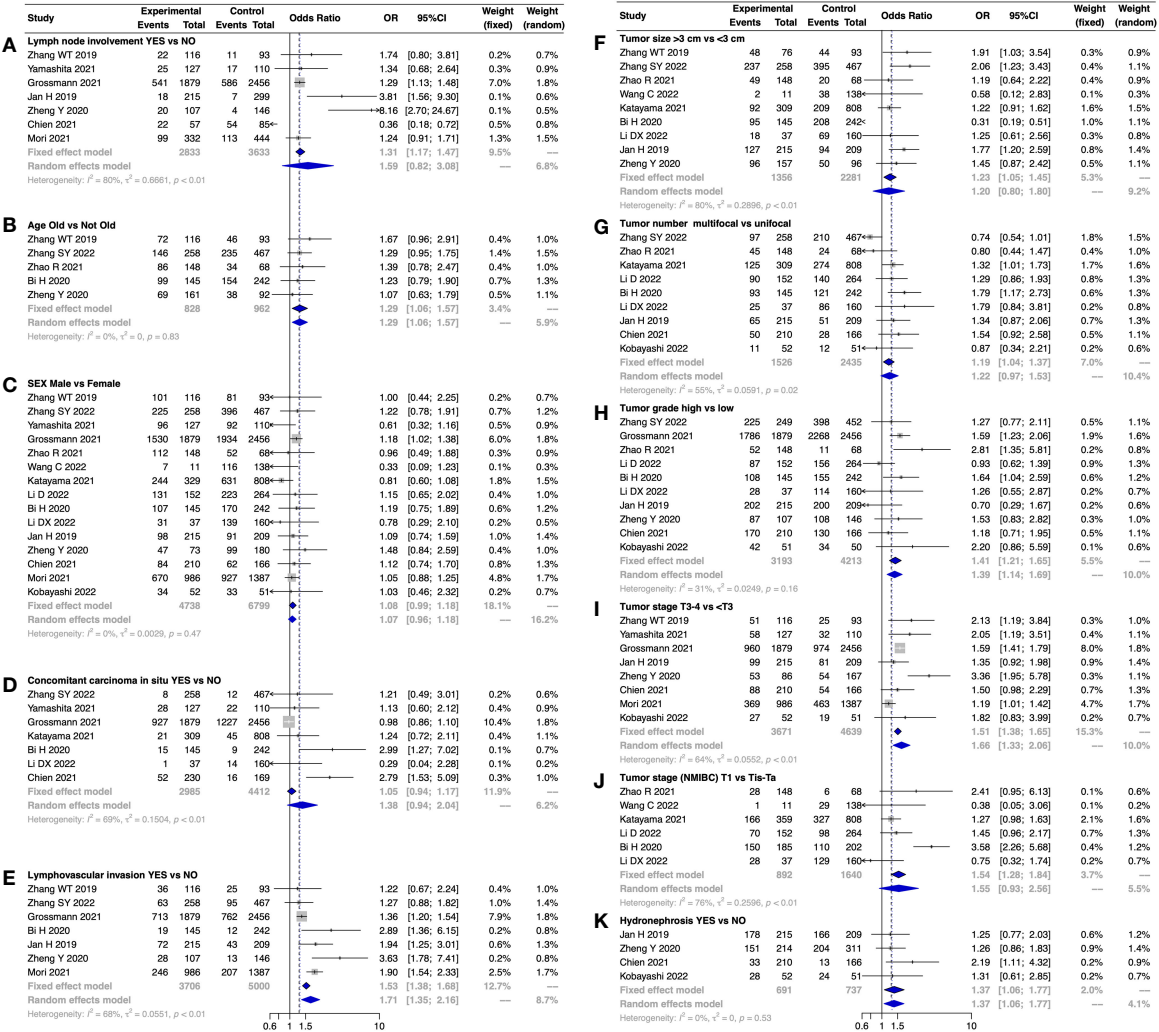
Forest plot showing the association between SII and clinicopathological features in patients with urothelial carcinoma. **(A)** lymph node involvement (yes vs. no); **(B)** age (old vs. not old); **(C)** sex (male vs. female); **(D)** concomitant carcinoma *in situ* (yes vs. no); **(E)** lymphovascular invasion (yes vs. no); **(F)** tumor size (> 3 cm vs. < 3 cm); **(G)** tumor number (multifocal vs. unifocal); **(H)** tumor grade (high vs. low); **(I)** tumor stage (T3-4 vs. < T3); **(J)** tumor stage (NMIBC: T1 vs. Tis-Ta); and **(K)** hydronephrosis (yes vs. no).

We summarized the relationships between SII and 11 clinicopathological features in **Figure 4** and **Table 3**. Using a fixed-effects model, we found that high SII levels were significantly linked to advanced age (OR 1.29, 95% CI 1.06–1.57, I^2^ = 0%) and high tumor grade (OR 1.41, 95% CI 1.21–1.65, I^2^ = 31%). Additionally, a random-effects model showed that increased SII levels were significantly associated with lymphovascular invasion (OR 1.71, 95% CI 1.35–2.16, I^2^ = 68%) and high tumor stage (OR 1.66, 95% CI 1.33–2.06, I^2^ = 0%). Moreover, SII was found to be positively related to hydronephrosis in UTUC patients undergoing RNU (OR 1.37, 95% CI 1.06–1.77, I^2^ = 0%) (**Table 3**).

**Table 3:** The meta-analysis of association between SII and clinicopathological factors in patients with urothelial carcinoma.

Variables	No. of studies	No. of patients	OR (95% CI)	*I^2^* (%)	*P* for heterogeneity	Model
Age (old vs not old)	5	1790	**1.29 (1.06-1.57)**	0	0.83	Fixed
Sex (male vs female)	15	11537	1.08 (0.99-1.18)	0	0.47	Fixed
Concomitant carcinoma *in situ* (yes vs no)	7	7397	1.38 (0.94-2.04)	69	<0.01	Random
Lymph node involvement (yes vs no)	7	6466	1.59 (0.82-3.08)	80	<0.01	Random
Lymphovascular invasion (yes vs no)	7	8706	**1.71 (1.35-2.16)**	68	<0.01	Random
Tumor size (> 3cm vs < 3cm)	9	3637	1.20 (0.80-1.80)	80	<0.01	Random
Tumor number (multifocal vs unifocal)	9	3961	1.22 (0.97-1.53)	55	0.02	Random
Tumor grade (high vs low)	10	7406	**1.41 (1.21-1.65)**	31	0.16	Fixed
Tumor stage (T3-4 vs < T3)	8	8310	**1.66 (1.33-2.06)**	64	<0.01	Random
Tumor stage (NMIBC: T1 vs Tis-Ta)	6	2532	1.55 (0.93-2.56)	76	<0.01	Random
Hydronephrosis (yes vs no)	4	1428	**1.37 (1.06-1.77)**	0	0.53	Fixed

HR, hazard ratio; CI, confidence interval; SII, systemic immune-inflammation index; MIBC, muscle invasive bladder cancer; NMIBC, non-muscle invasive bladder cancer; UTUC, upper urinary tract urothelial carcinoma.

Bold value: There is a significant correlation between SII and this clinicopathological factor.

### 3.7 Sensitivity analysis and publication bias

We conducted a sensitivity analysis by omitting each study one by one and found that the pooled HRs of OS, CSS, and PFS did not change significantly, indicating the reliability and robustness of the results (**Supplementary Figures 1A–C**). To assess publication bias, we performed the Begg test and observed statistically significant P-values in OS (P=0.03) and CSS (P=0.02), but not in RFS (P=0.08). Thus, we used the trim and fill method to adjust the results in OS and CSS and still found a positive association between SII and OS and CSS, with HRs of 1.31 (95%CI 1.11-1.54) and 1.34 (95% CI 1.05-1.72), respectively (**Supplementary Figures 2A, B**), confirming the prognostic value of SII.

## 4 Discussion

To our knowledge, this is the first meta-analysis to comprehensively examine the association between SII levels and UC based on 20 studies. Our findings demonstrated elevated SII levels as an independent prognostic biomarker for OS, CSS, and RFS in patients with UC. Moreover, our meta-analysis provided novel evidence indicating that elevated SII levels were associated with adverse prognosis in UTUC patients receiving RNU, including OS, CSS, and RFS. Furthermore, SII predicted the response to intravesical BCG treatment and disease recurrence in patients with NMIBC receiving TURBT and BCG immunotherapy. High SII levels were associated with advanced age, lymphovascular invasion, hydronephrosis, and high tumor grade and stage.

Many systematic reviews and meta-analyses have effectively synthesized the findings from multiple studies, yielding invaluable insights into the intricate relationship between SII and solid tumors such as breast (33), nasopharyngeal (34), colorectal (35), lung (36), prostate (9), and pancreatic cancer (37). The collective evidence consistently indicates a noteworthy association, demonstrating that elevated SII levels significantly correlate with unfavorable prognosis in patients afflicted with these specific cancer types. In addition, SII levels have been found to independently predict the prognosis of patients with BC undergoing RC or TURBT. In a meta-analysis of 10 studies, Li J et al. reported that elevated SII levels were associated with diminished OS rate (HR = 1.22, I^2^ = 81.1), CSS rate (HR = 1.68, I^2^ = 73.6%), and RFS rate (HR = 1.29, I^2^ = 90.2%) in BC patients, with high heterogeneity (38). Moreover, prior studies have demonstrated the correlation between adverse prognosis in bladder cancer and both the NLR and PLR (39–41). By combining the counts of neutrophils, lymphocytes, and platelets, SII integrates these constituent factors and effectively reflects their involvement in NLR and PLR, thereby establishing a comprehensive link between inflammation-related markers and bladder cancer prognosis. Considering that patients undergoing RC and TURBT represent different disease states, we conducted separate meta-analyses for each treatment type. In RC-treated BC patients, we found that the pooled outcomes unveiled a significant correlation between SII and OS, CSS, and RFS. Likewise, in TURBT-treated BC patients, elevated SII levels were associated with increased tumor-specific death and disease recurrence. Despite the observed heterogeneity attributed to sample size, a consistent relationship between SII and both OS and RFS was evident within subgroups stratified by sample size, thus further affirming the robust relationship between SII and the prognosis of bladder cancer patients.

Despite previous studies reporting a relationship between SII and survival outcomes in UTUC patients, a comprehensive meta-analysis is currently lacking. A large multicenter study demonstrated that preoperative SII was significantly associated with worse OS, CSS, and RFS in UTUC patients who underwent RNU (28). Another study found that high SII levels increased the risk of all-cause and cancer-specific mortality but did not affect disease progression. They also showed that combining SII and MLR could better predict disease progression and survival (29). Similarly, Zheng Y et al. revealed that the combination of SII and prognostic nutritional index was a strong independent predictor of UTUC patients receiving RNU (12). Furthermore, Chien et al. not only examined the positive association of high SII levels with metastasis and cancer-specific mortality, but also with bladder recurrence after RNU (10). Based on these studies, our meta-analysis, for the first time, found that increased preoperative SII independently increased the risk of OS, CSS, and RFS in UTUC patients after RNU. These findings provide valuable insights into the use of SII as a prognostic marker in UTUC patients, which could guide the application of neoadjuvant chemotherapy and lymph node dissection in RNU.

TURBT and intravesical BCG therapy are recommended for intermediate- and high-risk NMIBC patients to decrease the risk of recurrence and progression of NMIBC (16, 18). However, BCG non-responders are unlikely to respond to further BCG therapy (42), highlighting the need for predictive indicators of BCG response and disease prognosis. Three studies had shown that SII was a better predictor of response to intravesical BCG treatment in NMIBC than other inflammatory indexes, such as NLR and PLR, with a pooled AUC of 0.74 (19, 22, 23). Moreover, both Ke ZB et al. and Liu P et al. found that preoperative SII was an independent predictor of BCG treatment response using multivariate logistic regression analysis (19, 22). Thus, we combined these results and concluded that high SII levels independently increased the risk of BCG-unresponsive in NMIBC. Furthermore, we found that SII was not only associated with prognosis in patients undergoing RC or RNU, but also a vital predictor of NMIBC recurrence after TURBT and intravesical BCG treatment. The European Organization for Research and Treatment of Cancer (EORTC) risk score is used to estimate the risk of recurrence and progression in NMIBC after TURBT and intravesical BCG therapy (42). Liu P et al. reported that combining SII with the EORTC system improved the accuracy of predicting recurrence (19). Notably, these studies were small retrospective studies, and large-scale prospective studies are needed to confirm the association between SII and BCG response in NMIBC patients. In summary, predicting BCG failure might help avoid ineffective BCG therapy and facilitate timely implementation of RC.

SII has been widely used as a practical, cost-effective, and non-invasive biomarker for predicting the adverse prognosis in tumors (43, 44). The predictive role of SII in cancer could be explained by the several underlying mechanisms. Firstly, cancer patients are known to exhibit a hypercoagulable state, as cancer cells can induce platelet activation leading to an increased risk of thrombotic events (45). Additionally, platelets can promote the growth of angiogenic-dependent tumors by upregulating the levels of VEGF, TGF-β, FDGF, PF4, and IL-6 (46–48). Secondly, tumor-associated neutrophils play an important role in the tumor microenvironment, contributing to angiogenesis, matrix remodeling, metastasis and immunosuppression, all of which contribute to the tumor formation and development (49, 50). Furthermore, lymphocytes are considered as a crucial role in immune defense by promoting cytotoxic cell death and inhibiting tumor cell proliferation and migration (51). Therefore, high SII levels, resulting from elevated platelet and neutrophil counts and decreased lymphocyte counts, are frequently associated with poor prognosis for cancer patients.

This meta-analysis has several limitations that should be acknowledged. First, as all studies included were retrospective, there might be confounding factors that were not accounted for, affecting the accuracy of our findings. Further research should incorporate prospective studies to validate the current findings and generate more robust evidence. Second, the criteria for selecting cut-off values for SII varied among studies, potentially introducing selection bias related to factors such as race or clinical setting. However, most cut-off values of SII were usually around 500 (20), and in this study, the interquartile range of the cut-off values of SII were 467.76 to 610. Giving careful thought to standardization methods or offering a selection of cut-off values tailored to different patient populations or clinical settings holds significant merit. Third, publication bias needs to be considered. Despite the use of the trim and fill method to adjust the results in OS and CSS, the positive association between SII and OS and CSS remained unchanged, thereby confirming the prognostic value of SII. Finally, significant heterogeneity was found for OS, CSS, and RFS. However, we performed subgroup analysis and found the potential origin of heterogeneity. SII remained significantly related to OS and RFS in both subgroups of sample size.

## 5 Conclusions

This meta-analysis reveals that elevated SII levels are significantly associated with worse OS, CSS, and RFS in patients with UC, including those with UTUC receiving RNU. Furthermore, SII shows promise in predicting the response to intravesical BCG immunotherapy and disease recurrence in patients with NMIBC. As a cost-effective and practical biomarker, SII could serve as a useful tool for guiding the management and follow-up of UC patients, especially those with NMIBC receiving intravesical BCG therapy. However, further large-scale prospective validation studies are necessary to confirm these findings.

## Data availability statement

The original contributions presented in the study are included in the article/**Supplementary Material**. Further inquiries can be directed to the corresponding author.

## Author contributions

Material preparation, data collection and analysis were performed by WL, YZ, MMW, and MW. The draft of the manuscript was written by WL and YZ. The manuscript was revised by MMW and QY. All authors contributed to the article and approved the submitted version.

## Supplementary material

Supplementary Figure 1 Sensitivity analysis of each included study. **(A)** OS for individual studies with sequential removal of each study; **(B)** CSS for individual studies with sequential removal of each study; **(C)** RFS for individual studies with sequential removal of each study. OS, overall survival; CSS, cancer-specific survival; RFS, recurrence-free survival.

Supplementary Figure 2 Funnel plots and publication bias assessment using Begg test and the trim and fill method. **(A)** filled funnel plot for OS; **(B)** filled funnel plot for CSS. OS, overall survival; CSS, cancer-specific survival.
