# Supplementary material for: High systemic immune-inflammation index predicts poor prognosis and response to intravesical BCG treatment in patients with urothelial carcinoma: a systematic review and meta-analysis
Source: Front Oncol. 2023 Nov 1;13:1229349. doi: 10.3389/fonc.2023.1229349 (PMC10646434; doi:10.3389/fonc.2023.1229349)
Supplement: Supplementary file 1 [file Table_1.docx]

| **Supplementary table 1. Quality evaluation of included cohort studies** | | | | |
| --- | --- | --- | --- | --- |
| Study | Selection | Comparability | Ootcomes | Sum |
| Zhang WT 2019 | ★★★★ | ★★ | ★ ★ | 8 |
| Zhang SY 2022 | ★★★★ | ★ | ★★★ | 8 |
| Ali Yılmaz 2020 | ★★★★ | ★★ | ★★★ | 9 |
| Hasan Yilmaz 2022 | ★★★★ | ★★ | ★ ★ | 8 |
| Yamashita 2021 | ★★★★ | ★★ | ★★★ | 9 |
| Grossmann 2021 | ★★★★ | ★★ | ★★★ | 9 |
| Zhao R 2021 | ★★★★ | ★ | ★★★ | 8 |
| Wang C 2022 | ★★★★ | ★★ | ★★★ | 9 |
| Katayama 2021 | ★★★★ | ★★ | ★★★ | 9 |
| Li D 2022 | ★★★★ | ★★ | ★★★ | 9 |
| Akan 2020 | ★★★★ | ★ | ★ ★ | 7 |
| Bi H 2020 | ★★★★ | ★★ | ★★★ | 9 |
| Li DX 2022 | ★★★★ | ★ | ★ ★ | 7 |
| Ke ZB 2021 | ★★★★ | ★★ | ★★★ | 9 |
| Liu P 2022 | ★★★★ | ★ | ★ | 6 |
| Jan H 2019 | ★★★★ | ★★ | ★★★ | 9 |
| Zheng Y 2020 | ★★★★ | ★ | ★ ★ | 7 |
| Chien 2021 | ★★★★ | ★ | ★★★ | 8 |
| Mori 2021 | ★★★★ | ★ | ★★★ | 8 |
| Kobayashi 2022 | ★★★★ | ★ | ★★ | 7 |
| NOTE. Selection: Representativeness of the exposed cohort, Selection of the non-exposed cohort, Ascertainment of exposure, Demonstration that outcome of interest was not present at start of study; Comparability: Comparability of cohorts on the basis of the design or analysis; Outcomes: Assessment of outcome, Was follow-up long enough for outcomes to occur?, Adequacy of follow up of cohort. | | | | |
