# Supplementary figures and images for: High systemic immune-inflammation index predicts poor prognosis and response to intravesical BCG treatment in patients with urothelial carcinoma: a systematic review and meta-analysis
Source: Front Oncol. 2023 Nov 1;13:1229349. doi: 10.3389/fonc.2023.1229349 (PMC10646434; doi:10.3389/fonc.2023.1229349)

(a) Overall survival

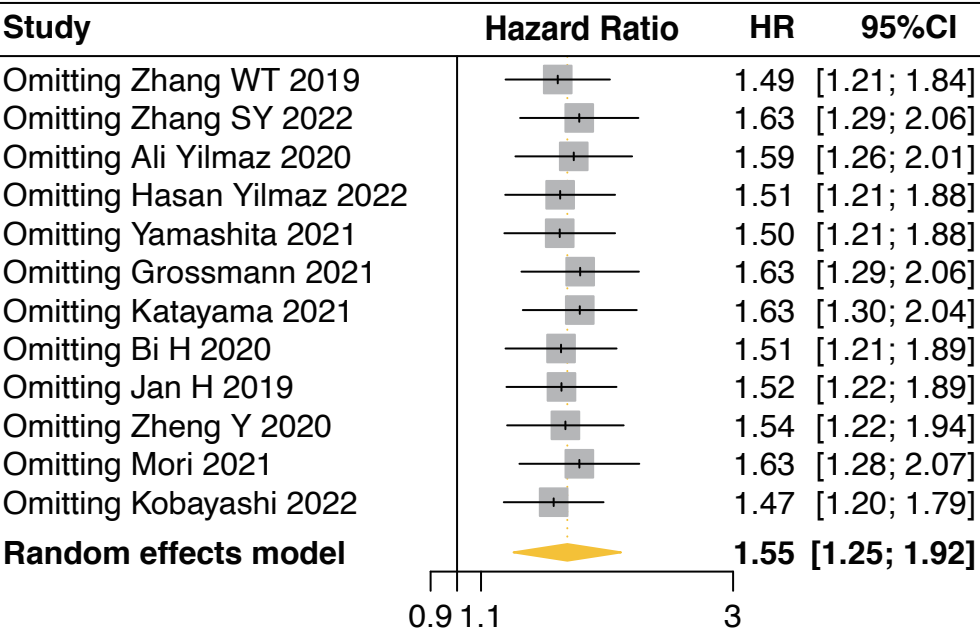

(b) Cancer-specific survival

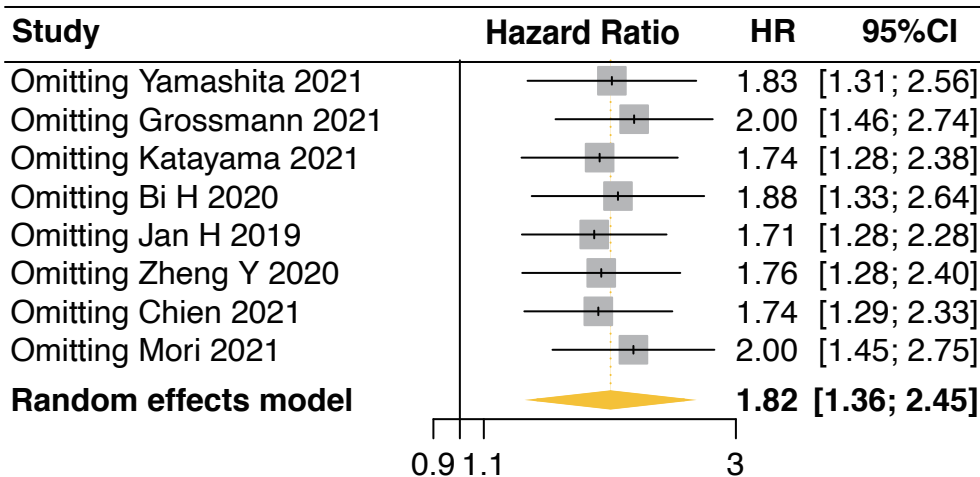

(c) Recurrence-free survival

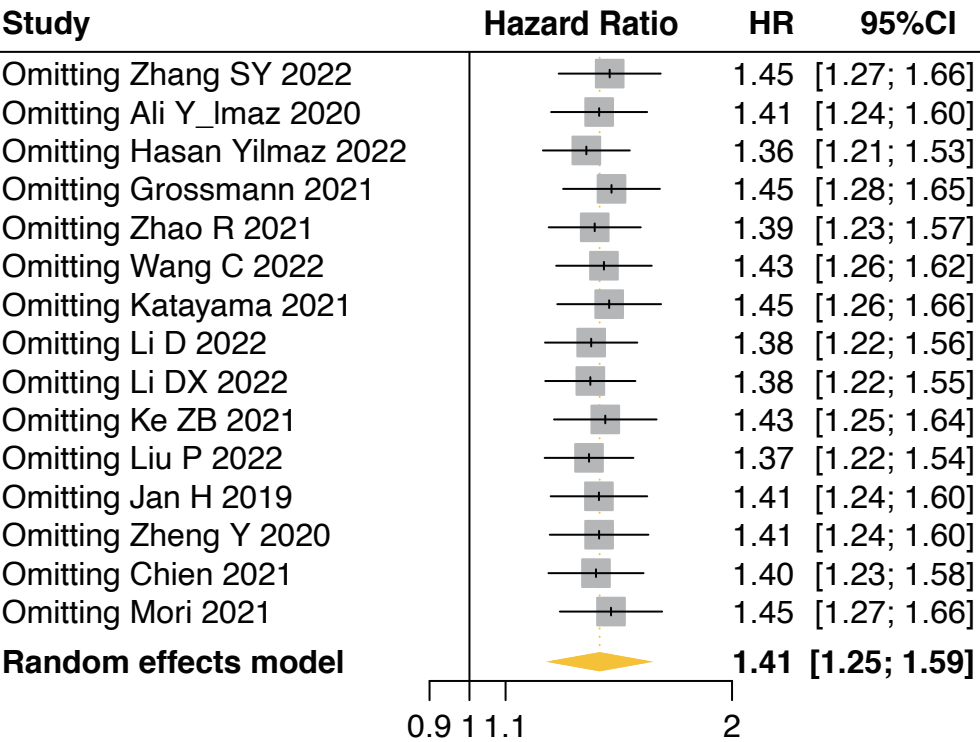

Supplement: Supplementary Figure 1 — Sensitivity analysis of each included study. (A) OS for individual studies with sequential removal of each study; (B) CSS for individual studies with sequential removal of each study; (C) RFS for individual studies with sequential removal of each study. OS, overall survival; CSS, cancer-specific survival; RFS, recurrence-free survival. [file DataSheet_1.pdf]
