# Supplementary material for: High systemic immune-inflammation index predicts poor prognosis and response to intravesical BCG treatment in patients with urothelial carcinoma: a systematic review and meta-analysis
Source: Front Oncol. 2023 Nov 1;13:1229349. doi: 10.3389/fonc.2023.1229349 (PMC10646434; doi:10.3389/fonc.2023.1229349)
Supplement: Supplementary Figure 2 — Funnel plots and publication bias assessment using Begg test and the trim and fill method. (A) filled funnel plot for OS; (B) filled funnel plot for CSS. OS, overall survival; CSS, cancer-specific survival. [file DataSheet_2.pdf]

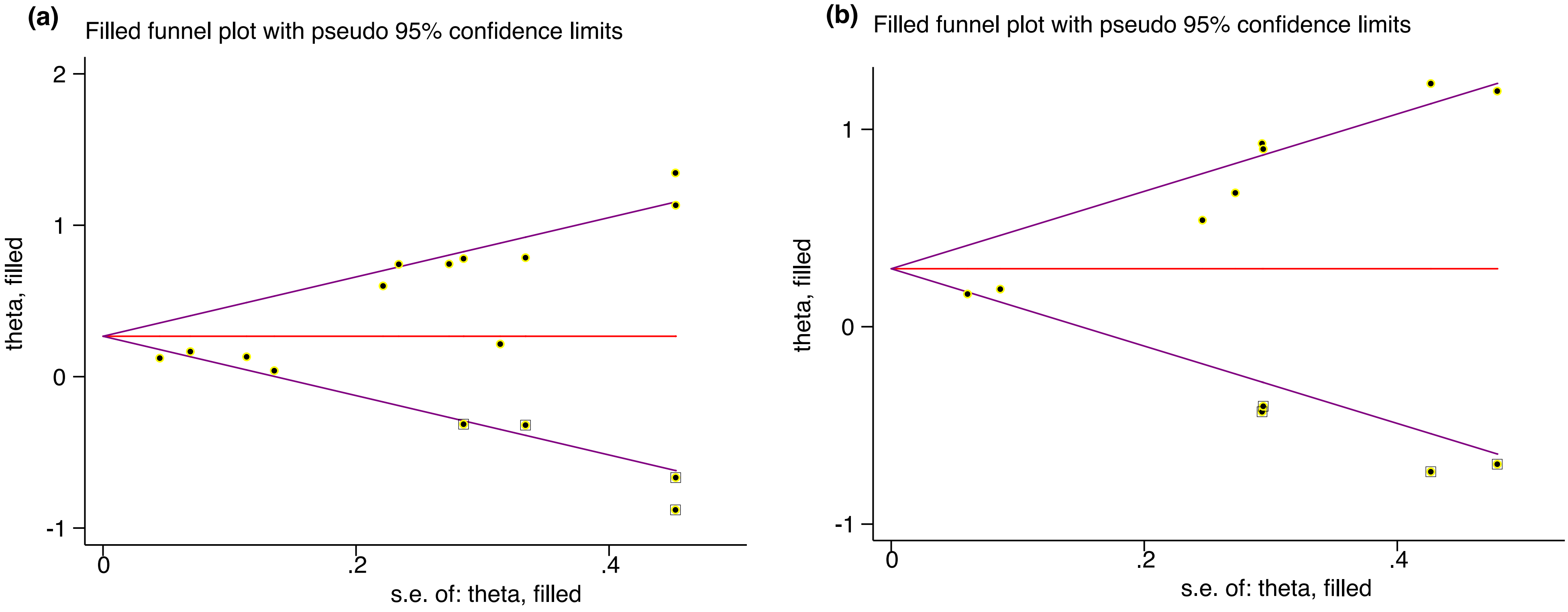
(a)
Filled funnel plot with pseudo 95% confidence limits
theta, filled
s.e. of: theta, filled
2
1
0
-1
0
.2
.4
(b)
Filled funnel plot with pseudo 95% confidence limits
theta, filled
s.e. of: theta, filled
1
0
-1
0
.2
.4
